# Glucagon-like peptide-1 receptor agonists: new strategies and therapeutic targets to treat atherosclerotic cardiovascular disease

**DOI:** 10.3389/fphar.2024.1396656

**Published:** 2024-04-24

**Authors:** Tianyu Wang, Juncan Ding, Xinyi Cheng, Qiang Yang, Pengfei Hu

**Affiliations:** ^1^ Department of The Second Clinical Medical College, Zhejiang Chinese Medical University, Hangzhou, China; ^2^ Department of Cardiology, The Second Affiliated Hospital of Zhejiang Chinese Medical University, Hangzhou, China

**Keywords:** glucagon-like peptide-1 receptor agonists, atherosclerosis, cardiovascular outcome trials, inflammation, mitochondrial dysfunction

## Abstract

Atherosclerotic cardiovascular disease (ASCVD) is a leading cause of cardiovascular mortality and is increasingly prevalent in our population. Glucagon-like peptide-1 receptor agonists (GLP-1RAs) can safely and effectively lower glucose levels while concurrently managing the full spectrum of ASCVD risk factors and improving patients’ long-term prognosis. Several cardiovascular outcome trials (CVOTs) have been carried out to further investigate the cardiovascular benefits of GLP-1RAs. Analyzing data from CVOTs can provide insights into the pathophysiologic mechanisms by which GLP-1RAs are linked to ASCVD and define the use of GLP-1RAs in clinical practice. Here, we discussed various mechanisms hypothesized in previous animal and preclinical human studies, including blockade of the production of adhesion molecules and inflammatory factors, induction of endothelial cells’ synthesis of nitric oxide, protection of mitochondrial function and restriction of oxidative stress, suppression of NOD-like receptor thermal protein domain associated protein three inflammasome, reduction of foam cell formation and macrophage inflammation, and amelioration of vascular smooth muscle cell dysfunction, to help explain the cardiovascular benefits of GLP-1RAs in CVOTs. This paper provides an overview of the clinical research, molecular processes, and possible therapeutic applications of GLP-1RAs in ASCVD, while also addressing current limitations in the literature and suggesting future research directions.

## 1 Introduction

Atherosclerotic cardiovascular disease (ASCVD) encompasses a range of conditions, comprising acute coronary syndrome (ACS), stable angina pectoris, coronary or other revascularization, transient ischemic attack, ischemic stroke, and peripheral vascular lesions (Bostrom et al., 2021). The disease process of atherosclerosis (AS) is intricate, involving the accumulation of AS-causing lipoproteins in endothelial cells and the formation of AS plaques due to extracellular matrix, vascular smooth muscle cells (VSMCs), immune cells, immunoglobulins, necrotic cellular debris, and neovascularization with intra-plaque hemorrhage (Libby et al., 2019). ASCVD accounted for over 17 million fatalities worldwide in 2015 (Belardo et al., 2022; Liu et al., 2022), underscoring its significant impact on individuals, their families, and social healthcare systems, and highlighting the urgent need for preventive and therapeutic measures for ASCVD (Lenharo, 2023). Preventing the progression of severe ASCVD is expected to reduce the occurrence of major adverse cardiovascular events (MACE) (Kalra et al., 2021).

Risk factors for ASCVD include hypertension, hyperglycemia, hyperlipidemia, smoking, and obesity (Chu et al., 2020). While progress has been made in reducing the incidence of ASCVD through smoking cessation, blood pressure and low-density lipoprotein (LDL) control, the global rise in type 2 diabetes mellitus (T2DM) poses a new challenge for the prevention and treatment of ASCVD (Wang et al., 2018). T2DM is a highly influential risk factor for the progression of ASCVD (Valanti et al., 2021). With the implementation of standard treatments for T2DM, including weight loss, glucose reduction, as well as antihypertensive and lipid-lowering therapies, patients with T2DM have had a notable decrease in both cardiovascular events and mortality. However, there is still much room for improvement, as cardiovascular issues remain a leading cause of mortality among patients with T2DM (Acosta et al., 2021). Glucagon-like peptide-1 (GLP-1) is an intestinal hormone that acts as an insulin stimulator. It promotes the release of insulin from pancreatic β-cells in response to glucose while inhibiting glucagon secretion from pancreatic α-cells. Furthermore, GLP-1 slows stomach emptying and helps convey feelings of fullness in the central nervous system (Scheen, 2018; Tilinca et al., 2021). The GLP-1 receptor (GLP-1R) is widely distributed in the cardiovascular system. Multinational, multicenter prospective cardiovascular outcome trials (CVOTs) have verified that GLP-1R agonists (GLP-1RAs) provide cardiovascular benefits and reduce cardiovascular mortality independent of their glucose-lowering effects (Caruso et al., 2019).

This paper provides a comprehensive review of CVOTs investigating the use of GLP-1RAs in individuals with risk factors for or diagnosed with ASCVD, offering critical data from these CVOTs. In addition, this paper also make investigation into the direct relationship between GLP-1RAs and AS pathogenesis.

## 2 Methods

**FIGURE 1:**
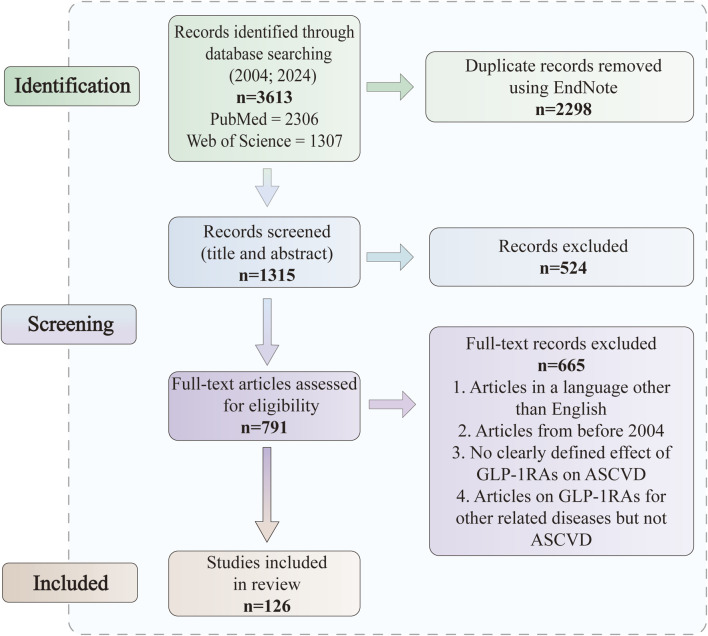
PRISMA flow diagram of included studies.

We searched PubMed and Web of Science for articles published between February 2004 and February 2024. We searched the literature according to the Preferred Reporting Items for Systematic Reviews and Meta-Analyses (PRISMA) guidelines (Page et al., 2021). The search terms were “glucagon-like peptide-1”, “glucagon-like peptide-1 receptor agonist”, “beinaglutide”, “dulaglutide”, “exenatide”, “semaglutide”, “liraglutide”, “type 2 diabetes mellitus”, “atherosclerosis”, “atherosclerotic cardiovascular disease”, “coronary atherosclerosis”, “stable angina”, “unstable angina”, “coronary revascularization”, “cardiovascular outcome trials”, “platelet activation”, “blood glucose”, “blood pressure, lipids”, “macrophages”, “inflammation” and “oxidative stress” alone or in combination. Inclusion and exclusion criteria were used in the selection of articles (Figure 1).

### 2.1 Inclusion

• all types of ASCVD
• qualifying both *in vivo* and *in vitro* studies

### 2.2 Exclusion

• articles in a language other than English
• articles from before 2004
• no clearly defined effect of GLP-1RAs on ASCVD
• articles on GLP-1RAs for other related diseases but not ASCVD

## 3 GLP-1 and its receptors

Following food intake, particularly glucose and other carbs, neuroendocrine L cells in the distal ileum and colon biphasically produce GLP-1, a peptide of 30 amino acids (Trujillo et al., 2021). Upon release into the bloodstream, GLP-1 attaches to and activates a specific and widely expressed receptor, GLP-1R (Aroda, 2018). Endogenous GLP-1 has a short plasma half-life of roughly two to 3 minutes (Chen et al., 2022). Consequently, GLP-1RAs have emerged to extend the half-life by modifying GLP-1’s structure and binding to GLP-1R in a glucose concentration-dependent manner to regulate blood glucose (Hall et al., 2018). Numerous clinical investigations have confirmed the hypoglycemic effectiveness of GLP-1RAs, which are categorized into short-acting and long-acting formulations. Short-acting GLP-1RAs, such as beinaglutide, lixisenatide, and exenatide, primarily reduce postprandial blood glucose and delay stomach emptying (Kaneto et al., 2021). In contrast, long-acting GLP-1RAs, including liraglutide, dulaglutide, semaglutide, and albiglutide, increase insulin secretion and decrease glucagon secretion, thus lowering fasting and postprandial blood glucose levels (Davies et al., 2018). GLP-1RAs have been demonstrated to offer significant cardiovascular benefits in addition to their glucose-lowering effects (Thomas et al., 2023). They prevent and stabilize ASCVD in both patients with and without T2DM through mechanisms that include not only glucose-lowering and weight-loss but also inhibition of inflammatory factors and adhesion molecules, suppression of the NOD-like receptor thermal protein domain associated protein 3 (NLRP3) inflammasome, induction of nitric oxide (NO) synthesis in endothelial cells, protection of mitochondrial function and inhibition of oxidative stress, reduction of macrophage inflammation and foam cell formation, and improvement of VSMC dysfunction (Sposito et al., 2018; Saraiva and Franco, 2021; Yang et al., 2021).

## 4 CVOTs of GLP-1RAs

The U.S. Food and Drug Administration published CVD risk evaluation guidelines for new drugs for T2DM in 2008 (Ussher and Drucker, 2023). GLP-1RAs have been demonstrated to offer significant cardiovascular benefits in addition to their glucose-lowering effects (Table 1). These studies were conducted to thoroughly examine the impact of the medication and placebo on cardiovascular outcomes in individuals with T2DM diagnosed with or having risk factors for CVD (Dardano et al., 2020). GLP-1RAs demonstrated superior glucose-lowering effects in CVOTs and significant effects on MACE risk reduction (Table 2), attracting considerable attention from cardiologists (Schubert et al., 2020; Martens et al., 2023).

**TABLE 1:** Effects of GLP-1RAs on change from baseline age (yr), BMI (kg/m^2^), HbA_1c_ (%), LDL-C (mg/dL), SBP (mmHg) and eGFR (ml/min/1.73 m^2^) in people with or without T2DM.

	Inclusion criteria	Drugs	N	Age	BMI	HbA_1c_	LDL-C	SBP	eGFR
ELIXA	T2DM with ACS in the preceding half a year	Lixisenatide 10–20 μg	3,034	59.9 ± 9.7	30.1 ± 5.6	7.7 ± 1.3	78.8 ± 35.4	129.0 ± 17.0	76.7 ± 21.3
		Placebo	3,034	60.6 ± 9.6	30.2 ± 5.8	7.6 ± 1.3	78.2 ± 35.2	130.0 ± 17.0	75.2 ± 21.4
LEADER	T2DM with high CVD risk or confirmed CVD	Liraglutide 1.8 mg	4,668	64.2 ± 7.2	32.5 ± 6.3	8.7 ± 1.6	NA	135.9 ± 17.8	80.2
		Placebo	4,672	64.4 ± 7.2	32.5 ± 6.3	8.7 ± 1.5		135.9 ± 17.7	80.6
SUSTAIN-6	T2DM with high CVD risk or confirmed CVD or CKD	Semaglutide 0.5 mg	826	64.6 ± 7.3	32.7 ± 6.3	8.7 ± 1.4	81.6 ± 47.1	136.1 ± 18.0	NA
		Semaglutide 1.0 mg	822	64.7 ± 7.1	32.9 ± 6.2	8.7 ± 1.5	83.3 ± 41.2	135.8 ± 17.0	
		Placebo 0.5 mg	824	64.8 ± 7.6	32.9 ± 6.4	8.7 ± 1.5	80.9 ± 48.1	135.8 ± 16.2	
		Placebo 1.0 mg	825	64.4 ± 7.5	32.7 ± 6.0	8.7 ± 1.5	83.6 ± 45.9	134.8 ± 17.5	
EXSCEL	T2DM with high CVD risk or confirmed CVD	Exenatide 2 mg	7,356	62.0 (56.0, 68.0)	31.8 (28.2, 36.2)	8.0 (7.3, 8.9)	NA	NA	76.6 (61.3, 92.0) 76.0 (61.0, 92.0)
		Placebo	7,396	62.0 (56.0, 68.0)	31.7 (28.2, 36.1)	8.0 (7.3, 8.9)			
HARMONY	T2DM with confirmed CVD	Abirutide 30–50 mg	4,731	64.1 ± 8.7	32.3 ± 5.9	8.8 ± 1.5	NA	134.8 ± 16.6 134.7 ± 16.5	79.1 ± 25.6 78.9 ± 25.4
		Placebo	4,732	64.2 ± 8.7	32.3 ± 5.9	8.7 ± 1.5			
REWIND	T2DM with high CVD risk or confirmed CVD	Dulaglutide 1.5 mg	4,949	66.2 ± 6.5	32.3 ± 5.7	7.3 ± 1.1	99.0 ± 37.9	137.1 ± 16.6	75.3 (61.6, 91.8)
		Placebo	4,952	66.2 ± 6.5	32.3 ± 5.8	7.4 ± 1.1	99.0 ± 37.9	137.3 ± 17.0	74.7 (61.2, 90.6)
PIONEER 6	T2DM with high CVD risk or confirmed CVD	Oral Semaglutide 14 mg	1,591	66.0 ± 7.0	32.3 ± 6.6	8.2 ± 1.6	77.0 ± 34.6	135.0 ± 18.0	74.0 ± 21.0
		Placebo	1,592	66.0 ± 7.0	32.3 ± 6.4	8.2 ± 1.6	79.0 ± 32.5	136.0 ± 18.0	74.0 ± 21.0
AMPLITUDE-O	T2DM with high CVD risk or confirmed CVD or CKD	Efpeglenatide 4 or 6 mg	2,717	64.6 ± 8.2	32.9 ± 6.2	8.9 ± 1.5	79.6 ± 37.7	135.1 ± 15.5	72.2 ± 21.9
		Placebo	1,359	64.4 ± 8.3	32.4 ± 6.0	8.9 ± 1.5	80.0 ± 37.3	134.4 ± 15.6	72.9 ± 23.3
SELECT	BMI ≥27 kg/m^2^ with high CVD risk or confirmed CVD without T2DM	Semaglutide 2.4 mg	8,803	61.6 ± 8.9	33.3 ± 5.0	5.8 ± 0.3	78.0 (61.0, 102.0)	131.0 ± 15.6	82.4 ± 17.5
		Placebo	8,801	61.6 ± 8.8	33.4 ± 5.0	5.8 ± 0.3	78.0 (61.0, 102.0)	130.9 ± 15.3	82.5 ± 17.3

Data are mean ± SD, or median (IQR), unless otherwise stated. ACS, acute coronary syndrome; BMI, body mass index; CKD, chronic kidney disease; CVD, cardiovascular disease; eGFR, estimated glomerular filtration rate; GLP-1RAs, glucagon-like peptide-1, receptor agonists; HbA_1c_, glycated haemoglobin A_1c_; IQR, interquartile range; LDL-C, low-density lipoprotein; N, number of participants; NA, not allowed at baseline; SBP, systolic blood pressure; SD, standard deviation; T2DM, type 2 diabetes mellitus.

**TABLE 2:** CVOTs evaluating GLP-1RAs in people with CVD (with or without T2DM).

	Author (Year)	Clinicaltrials.gov identifier	GLP-1RAs	Intervention	N	Median duration of follow-up	MACE	Additional findings
ELIXA	Pfeffer et al. (2015)(Pfeffer et al., 2015)	NCT01147250	Lixisenatide	Lixisenatide 10–20 μg sc once a day vs. Placebo	Randomized = 6068 (Lixisenatide, 3,034; Placebo, 3,034)	25 months	Lixisenatide group: 13.4%	-
							Placebo group: 13.2%	
							HR 1.02; 95%CI 0.89–1.17	
							*p* < 0.001 for non-inferiority	
							*p* = 0.81 for superiority	
LEADER	Marso et al. (2016b)	NCT01179048	Liraglutide	Liraglutide 1.8 mg sc once a day vs. Placebo	Randomized = 9,340 (Liraglutide, 4,668; Placebo, 4,672)	3.8 years	Liraglutide group: 13.0%	Decrease in CV death in the group taking liraglutide (4.7% vs. 6.0%; HR 0.78; 95% CI 0.66–0.93; *p* = 0.007) Decrease in all-cause mortality in the group taking liraglutide (8.2% vs. 9.6%; HR 0.85; 95% CI 0.74–0.97; *p* = 0.02) Decrease in new-onset nephropathy or exacerbation of nephropathy in the group taking liraglutide (5.7% vs. 7.2%; HR = 0.78; 95% CI 0.67–0.92; *p* = 0.003)
							Placebo group: 14.9%	
							HR 0.87; 95%CI 0.78–0.97	
							*p* < 0.001 for non-inferiority	
							*p* = 0.01 for superiority	
SUSTAIN-6	; Marso et al., 2016a)	NCT01720446	Semaglutide	Semaglutide 0.5 or 1.0 mg sc once a week vs. Placebo	Randomized = 3,297 (Semaglutide, 1,648; Placebo, 1,649)	104 weeks	Semaglutide group: 6.6% Placebo group: 8.9% HR 0.74; 95% CI 0.58–0.95 *p* < 0.001 for non-inferiority *p* = 0.02 for superiority	Decrease in nonfatal stroke in the group taking semaglutide (1.6% vs. 2.7%; HR 0.61; 95% CI 0.38–0.99; *p* = 0.04)
								Decrease in developing new or worsening nephropathy in the group taking semaglutide (3.8% vs. 6.1%; HR = 0.64; 95% CI 0.46–0.88; *p* = 0.005)
EXSCEL	Holman et al. (2017)(Holman et al., 2017)	NCT01144338	Exenatide	Exenatide 2 mg sc once a week vs. Placebo	Randomized = 14752 (Exenatide, 7,356; Placebo, 7,396)	3.2 years	Exenatide group: 11.4%	-
							Placebo group: 12.2%	
							HR 0.91; 95% CI 0.83–1.00	
							*p* < 0.001 for non-inferiority	
							*p* = 0.06 for superiority	
HARMONY	Hernandez et al. (2018)(Hernandez et al., 2018)	NCT02465515	Abirutide	Abirutide 30–50 mg sc once a week vs. Placebo	Randomized = 9,463 (Abirutide, 4,731; Placebo, 4,732)	1.6 years	Abirutide group: 7.0%	-
							Placebo group: 9.0%	
							HR 0.78; 95% CI 0.68–0.90; *p* < 0.0001 for non-inferiority	
							*p* = 0.0006 for superiority	
REWIND	Gerstein et al. (2019)(Gerstein et al., 2019)	NCT01394952	Dulaglutide	Dulaglutide 1.5 mg sc once a week vs. Placebo	Randomized = 9,901 (Dulaglutide, 4,949; Placebo, 4,952)	5.4 years	Dulaglutide group: 12.0%	Decrease in nonfatal stroke in the group taking dulaglutide (2.7% vs. 3.5%; HR 0.76; 95% CI 0.61–0.95; *p* = 0.017)
							Placebo group: 13.4%	
							HR 0.88; 95% CI 0.79–0.99; *p* = 0.026	
PIONEER 6	Husain et al. (2019)(Husain et al., 2019)	NCT02692716	Oral Semaglutide	Oral Semaglutide 14 mg once a day vs. Placebo	Randomized = 3,183 (Oral Semaglutide, 1,591; Placebo, 1,592)	15.9 months	Oral Semaglutide group: 3.8% Placebo group: 4.8%	Decrease in CV death in the group taking oral semaglutide (0.9% vs. 1.9%; HR 0.49; 95% CI 0.27–0.92) Decrease in all-cause mortality in the group taking oral semaglutide (1.4% vs. 2.8%; HR 0.51; 95% CI 0.31–0.84)
							HR 0.79; 95% CI 0.57–1.11; *p* < 0.001 for non-inferiority	
							*p* = 0.17 for superiority	
AMPLITUDE-O	Gerstein et al. (2021)(Gerstein et al., 2021)	NCT03496298	Efpeglenatide	Efpeglenatide 4 or 6 mg sc once a week vs. Placebo	Randomized = 4,076 (Efpeglenatide, 2,717; Placebo, 1,359)	1.81 years	Efpeglenatide group: 7.0%	Decrease in composite renal outcome event in the group taking efpeglenatide (13.0% vs. 18.4%; HR 0.68; 95% CI 0.57–0.79; *p* < 0.001)
							Placebo group: 9.2%	
							HR 0.73; 95% CI 0.58–0.92; *p* < 0.001 for non-inferiority	
							*p* = 0.007 for superiority	
SELECT	Lincoff et al. (2023)(Lincoff et al., 2023)	NCT03574597	Semaglutide	Semaglutide 2.4 mg sc once a week vs. Placebo	Randomized = 17604 (Semaglutide, 8,803; Placebo, 8,801)	39.8 months	Semaglutide group: 6.5% Placebo group: 8.0% HR 0.80; 95% CI 0.72–0.90 *p* < 0.001	Decrease in CV death in the group taking semaglutide (2.5% vs. 3.0%; HR 0.85; 95% CI 0.71–1.01; *p* = 0.07)
								Decrease in heart failure composite end point in the group taking semaglutide (3.4% vs. 4.1%; HR 0.82; 95% CI 0.71–0.96)
								Decrease in all-cause mortality in the group taking semaglutide (4.3% vs. 5.2%; HR 0.81; 95% CI 0.71–0.93)

ACS, acute coronary syndrome; BMI, body mass index; CI, confidence interval; CKD, chronic kidney disease; CV, cardiovascular; CVD, cardiovascular disease; CVOTs, cardiovascular outcomes; GLP-1RAs, glucagon-like peptide-1, receptor agonists; HR, hazard ratio; MACE, major adverse cardiovascular event; N, number of participants; P, probability; Sc, subcutaneous; T2DM, type 2 diabetes mellitus; Vs., *versus*.

### 4.1 Elixa

The earliest CVOTs on GLP-1RAs were the ELIXA study (Al Rifai et al., 2022). The investigation included 6068 patients with T2DM who had suffered an ACS within the past 6 months. Patients were randomly allocated to the lixisenatide or placebo groups, receiving treatment with either drug (maximum dose of 20 μg once/day, with a beginning dose of 10 μg for 2 weeks), and a median follow-up of 25 months. The probability of four-point MACE was not much higher in the lixisenatide group than in the placebo group (13.4% vs. 13.2%; hazard ratio [HR] = 1.02; 95% confidence interval [CI] 0.89–1.17; *p* < 0.001 for non-inferiority; *p* = 0.81 for superiority), based on the findings. Similarly, there were no significant differences in individual endpoint comparisons. However, an analysis of other secondary endpoints showed that treatment with lixisenatide led to a lower risk of developing new-onset massive albuminuria in patients with T2DM, slowed the progression of diabetic nephropathy in patients with massive albuminuria-associated T2DM, and demonstrated a positive impact on maintaining body weight (Pfeffer et al., 2015). ELIXA is the first reported randomized, double-blind, non-inferiority CVOTs of GLP-1RAs (Muskiet et al., 2018).

### 4.2 Leader

The 2016 LEADER trial was the first CVOT to report the cardioprotective benefits of GLP-1RAs, with liraglutide being the first GLP-1RA shown to have cardiovascular benefits (Buse et al., 2020). With a median follow-up of 3.8 years, 9,340 individuals with T2DM who had risk factors for or were diagnosed with CVD were included in the research. These patients were randomly assigned to the liraglutide (1.8 mg, once/day) or the placebo group. Patients in the liraglutide group were shown to have a 13% lower risk of three-point MACE compared to those in the placebo group (13.0% vs. 14.9%; HR = 0.87; 95% CI 0.78–0.97; *p* < 0.001 for non-inferiority; *p* = 0.01 for superiority). Interestingly, the randomized therapy for three-point MACE lasted 12–18 months until the Kaplan-Meier cumulative event curves started diverging, suggesting that the cardiovascular benefits of liraglutide may be mediated through its anti-AS effects (Verma et al., 2020). Secondary endpoint analyses showed that liraglutide significantly reduced the risk of cardiovascular death (4.7% vs. 6.0%; HR = 0.78; 95% CI 0.66–0.93; *p* = 0.007) and all-cause mortality (8.2% vs. 9.6%; HR = 0.85; 95% CI 0.74–0.97; *p* = 0.02) compared to placebo. Additionally, shown by other secondary endpoint analysis, there was a 22% decrease in the risk of new-onset nephropathy or exacerbation of nephropathy in the liraglutide group (5.7% vs. 7.2%; HR = 0.78; 95% CI 0.67–0.92; *p* = 0.003) (Marso et al., 2016b; Mann et al., 2017). The LEADER study made liraglutide the second hypoglycemic agent after the sodium-glucose cotransporter-2 inhibitor (SGLT2i), empagliflozin, to demonstrate cardiovascular benefits in randomized clinical trials, which was a significant breakthrough and source of inspiration for cardiologists and researchers, marking a landmark in the field (Kristensen et al., 2019).

### 4.3 Sustain-6

The purpose of the trial was to determine whether semaglutide was not less safe for the cardiovascular system than a placebo in individuals with T2DM (Aroda et al., 2019). A total of 3297 T2DM patients aged 50 years or older were enrolled, with 83% having confirmed CVD, chronic kidney disease, or both. The participants were assigned to receive either semaglutide (0.5 or 1.0 mg once per week) or placebo at random, and were followed for a median duration of 104 weeks. The findings demonstrated that, in comparison to placebo, individuals receiving semaglutide had a noticeably decreased risk of three-point MACE (6.6% vs. 8.9%; HR = 0.74; 95% CI 0.58–0.95; *p* < 0.001 for non-inferiority; *p* = 0.02 for superiority), with a notable decrease in the risk of nonfatal stroke (1.6% vs. 2.7%; HR = 0.61; 95% CI 0.38–0.99; *p* = 0.04) and a significant cardiovascular benefit. However, when compared to placebo, semaglutide did not lower the risk of cardiovascular death, nonfatal myocardial infarction (MI), or all-cause mortality. Furthermore, semaglutide dramatically decreased the risk of developing new or worsening nephropathy (3.8% vs. 6.1%; HR = 0.64; 95% CI 0.46–0.88; *p* = 0.005) (Marso et al., 2016a). Currently, subcutaneous injection formulations of semaglutide have been submitted for marketing in several countries worldwide, and their clinical efficacy is promising. Although generally well tolerated, semaglutide was associated with an increased risk of retina-related issues (3.0% vs. 1.8%; HR = 1.76; 95% CI 1.11–2.78; *p* = 0.02), necessitating careful consideration of its use in patients with comorbid retinopathy.

### 4.4 Exscel

The EXSCEL study, a randomized, double-blind, placebo-controlled clinical trial (Bethel et al., 2020), is the largest global clinical trial on GLP-1RAs. It was designed to evaluate the cardiovascular safety and efficacy of a weekly formulation of exenatide in patients with T2DM at all levels of CVD risk combined (Davis et al., 2022). The study enrolled 14,752 T2DM patients with HbA_1C_ of 6.5%–10.0%, of whom 73.1% had a history of prior cardiovascular events, and 26.9% had no prior cardiovascular events. Participants were randomized to receive either exenatide (2 mg/week) or placebo on top of conventional therapy, with a median follow-up of 3.2 years. The primary composite endpoints of the study were cardiovascular death, nonfatal MI, and nonfatal stroke. The results showed that the risk of three-point MACE in the exenatide group was not significantly different from that in the placebo group (11.4% vs. 12.2%; HR = 0.91; 95% CI 0.83–1.00; *p* < 0.001 for noninferiority; *p* = 0.06 for superiority), implying that exenatide is safe in patients with T2DM but does not provide additional cardiovascular benefits. However, compared with placebo, exenatide improved several cardiovascular risk factors, expressing in overall least-squares mean difference such as a 0.53% decrease in HbA_1C_, a 1.27 kg decrease in body weight, and a 1.57 mmHg decrease in systolic blood pressure. There were no significant differences in the risk of severe hypoglycemia, acute pancreatitis, pancreatic cancer, and medullary thyroid carcinoma between the exenatide group and the placebo group (Holman et al., 2017).

### 4.5 Harmony

The HARMONY study of abirutide, published in 2018, enrolled 9,463 T2DM patients aged 40 years or older with confirmed ASCVD (coronary heart disease, cerebrovascular disease, or peripheral arterial disease) in 28 countries, excluding those with severe CKD (Green et al., 2018). Patients were randomized to receive either abirutide (30–50 mg/week) or placebo in addition to standard treatment, with a median follow-up of 1.6 years. Results showed a 22% reduction in the risk of three-point MACE in the abirutide group compared with the placebo group (7.0% vs. 9.0%; HR = 0.78; 95% CI 0.68–0.90; *p* < 0.0001 for non-inferiority; *p* = 0.0006 for superiority). Meanwhile, the Kaplan-Meier cumulative event curves for three-point MACE showed gradual separation only after 8 months of randomized treatment, suggesting that its cardiovascular protective effect was mainly achieved through anti-AS mechanisms. Despite the short follow-up period, the study adds more evidence to support the hypothesis that GLP-1RAs improve cardiovascular prognosis in patients with T2DM. There were no significant differences in the rates of acute pancreatitis, pancreatic cancer, medullary thyroid carcinoma, and other serious adverse events between the abirutide and placebo groups (Home, 2019). The drug was launched in Europe and the United States in 2014 but was later withdrawn from the global market in 2018 for economic reasons. Although the drug has been discontinued, the study provides essential information for understanding the GLP-1RAs class of hypoglycemic agents (Hernandez et al., 2018).

### 4.6 Rewind

The REWIND study of dulaglutide, published in 2019, stood out from previous CVOTs as the longest-follow-up CVOTs in the lowest CVD-risk and baseline glycated hemoglobin A_1c_ (HbA_1c_) population (Webb et al., 2022). The research enrolled 9,901 T2DM patients aged 50 years or older with confirmed CVD or risk factors for CVD, with a baseline HbA_1c_ of 7.2%. After a follow-up of 5.4 years, participants were assigned to either the placebo group or the dulaglutide (1.5 mg, once/week) group at random. The dulaglutide group showed a considerably reduced risk of three-point MACE than the placebo group (12.0% vs. 13.4%; HR = 0.88; 95% CI 0.79–0.99; *p* = 0.026), and a notable decline in the risk of nonfatal stroke (2.7% vs. 3.5%; HR = 0.76; 95% CI 0.61–0.95; *p* = 0.017). Stratified analyses revealed a more significant benefit for the primary composite endpoint in those aged ≥66 years, with a Body Mass Index (BMI) ≥ 32 kg/m^2^, and in the European or Asia-Pacific populations. In addition, when assessed within subgroups, the intervention HR for MACE was similar independent of the patient’s history of CVD, indicating dulaglutide’s efficacy in preventing both primary and secondary CVD (Gerstein et al., 2019). This favorable cardiovascular protective effect raises the questions of whether GLP-1RAs may be utilized in individuals with T2DM as a main preventive measure for CVD, which is expected to become a future research trend (Scott, 2020). However, as compared to other GLP-1RAs, dulaglutide showed a higher frequency of gastrointestinal side effects such as nausea and vomiting, and acute pancreatitis occurred in 12 users in the clinical study of this drug. While this may be related to individual patient factors, it still raises safety concerns that need further confirmation.

### 4.7 Pioneer 6

The PIONEER six study was the first global CVOT on oral GLP-1RAs (Husain et al., 2020). The study enrolled 3,183 T2DM patients with confirmed CVD or risk factors for CVD. They were randomized into two groups, receiving either semaglutide (14 mg, once/day) or placebo, with a median follow-up of 15.9 months. The study’s findings failed to demonstrate that the oral semaglutide, as opposed to a placebo, substantially decreased the risk of MACE (3.8% vs. 4.8%; HR = 0.79; 95% CI 0.57–1.11; *p* < 0.001 for non-inferiority; *p* = 0.17 for superiority). There was also no significant difference in the risk of nonfatal MI and nonfatal stroke. However, in secondary endpoints, oral semaglutide did lower the risk of cardiovascular death (0.9% vs. 1.9%; HR = 0.49; 95% CI 0.27–0.92) and all-cause mortality (1.4% vs. 2.8%; HR = 0.51; 95% CI 0.31–0.84) in comparison to placebo. Furthermore, the effects of oral semaglutide on metabolic markers and safety showed that oral semaglutide reduced HbA_1c_, body weight, and systolic blood pressure. In terms of safety, it was consistent with previous GLP-1RAs’ level of safety, with no additional safety concerns identified (Husain et al., 2019). Despite not demonstrating cardiovascular benefits, the study showed that oral semaglutide did not raise the risk of MACE, supporting its stability and safety for clinical use (Bain et al., 2019).

### 4.8 Amplitude-O

SGLT2i and GLP-1RAs have been shown to mechanistically complement each other in reducing cardiovascular events in patients with T2DM (Lam et al., 2022). The AMPLITUDE-O study was designed to evaluate the effect of SGLT2i on the efficacy and safety of efpeglenatide. Efpeglenatide is an animal-derived GLP-1RA based on the structure of exendin-4 with an elimination half-life of approximately 155 h (Tommerdahl et al., 2022). The study included 4,076 subjects with a baseline HbA_1c_ of 8.9%, of whom 31.6% had an estimated glomerular filtration rate (eGFR) of <60 mL/min/1.73 m^2^, 89.5% had comorbid CVD, and 15.0% were on SGLT2i. Subjects were randomized in a 1:1:1 ratio to three groups. They received 4 mg or 6 mg efpeglenatide or placebo weekly subcutaneously, respectively, with a median follow-up of 1.81 years. The primary composite endpoints of the study were nonfatal MI, nonfatal stroke, and death from cardiovascular causes or unknown causes. Although a large proportion of patients in the AMPLITUDE-O trial were also treated with SGLT2i, efpeglenatide reduced the risk of three-point MACE regardless of SGLT2i treatment. The risk of three-point MACE was significantly reduced by 27% in the efpeglenatide group compared with the placebo group (7.0% vs. 9.2%; HR = 0.73; 95% CI 0.58–0.92; *p* < 0.001 for non-inferiority; *p* = 0.007 for superiority). There was a significant 35% reduction in the risk of three-point MACE in the efpeglenatide 6 mg group and also a trend toward a benefit in the risk of three-point MACE in the efpeglenatide 4 mg group. Both efpeglenatide doses showed a possible quantity-effect relationship on the primary composite endpoint. The secondary endpoint showed a 32% reduction in the composite renal outcome event in the efpeglenatide group compared with the placebo group (13.0% vs. 18.4%; HR = 0.68; 95% CI 0.57–0.79; *p* < 0.001) (Gerstein et al., 2021). The graded benefit relationship between efpeglenatide dose and CVOTs suggests that using high doses of efpeglenatide or other GLP-1 RAs may maximize the cardiovascular and renal benefit profile (Gerstein et al., 2023).

### 4.9 Select

All of the above-mentioned CVOTs were conducted in patients with risk factors for or diagnosed CVD with comorbid T2DM. In contrast, the SELECT research was the first randomized, double-blind, multicenter, phase III clinical trial in patients with CVD who were not suffering from T2DM (Irfan, 2024). Prior to the SELECT study, there was no significant evidence of any hypoglycemic agents in reducing the risk of MACE or improving prognosis in individuals with CVD without T2DM (Nauck and Müller, 2023). The research recruited 17,604 overweight or obese individuals aged 45 years or older with confirmed CVD or high risk of CVD, excluding those with a HbA_1c_ ≥ 6.5% or confirmed T2DM. Participants were randomized to receive either semaglutide or placebo in addition to their regular therapy. A weekly beginning dosage of 0.24 mg of semaglutide was given, with dose escalations (0.5, 1.0, 1.7, and 2.4 mg/week) every 4 weeks until reaching the target dosage of 2.4 mg at week 16. According to the results, individuals treated with semaglutide exhibited a 20% reduction in experiencing a three-point MACE than those in the placebo group (6.5% vs. 8.0%; HR = 0.80; 95% CI 0.72–0.90; *p* < 0.001), and this reduction was consistent across subgroup analyses. Additionally, the semaglutide group had a 15% lower risk of cardiovascular death than the placebo group (2.5% vs. 3.0%; HR = 0.85; 95% CI 0.71–1.01; *p* = 0.07), an 18% reduced risk in the heart failure composite endpoint (3.4% vs. 4.1%; HR = 0.82; 95% CI 0.71–0.96), and a 19% lower risk of all-cause mortality (4.3% vs. 5.2%; HR = 0.81; 95% CI 0.71–0.93) according to secondary endpoint findings (Lincoff et al., 2023). The results of the SELECT study are expected to influence the guidelines for treating ASCVD, potentially leading to a paradigm shift in clinical practice and a broader application of other GLP-1RAs (Lingvay et al., 2023).

## 5 Molecular mechanism of anti-AS by GLP-1RAs

### 5.1 Inhibition of the expression of inflammatory factors and adhesion molecules

**FIGURE 2:**
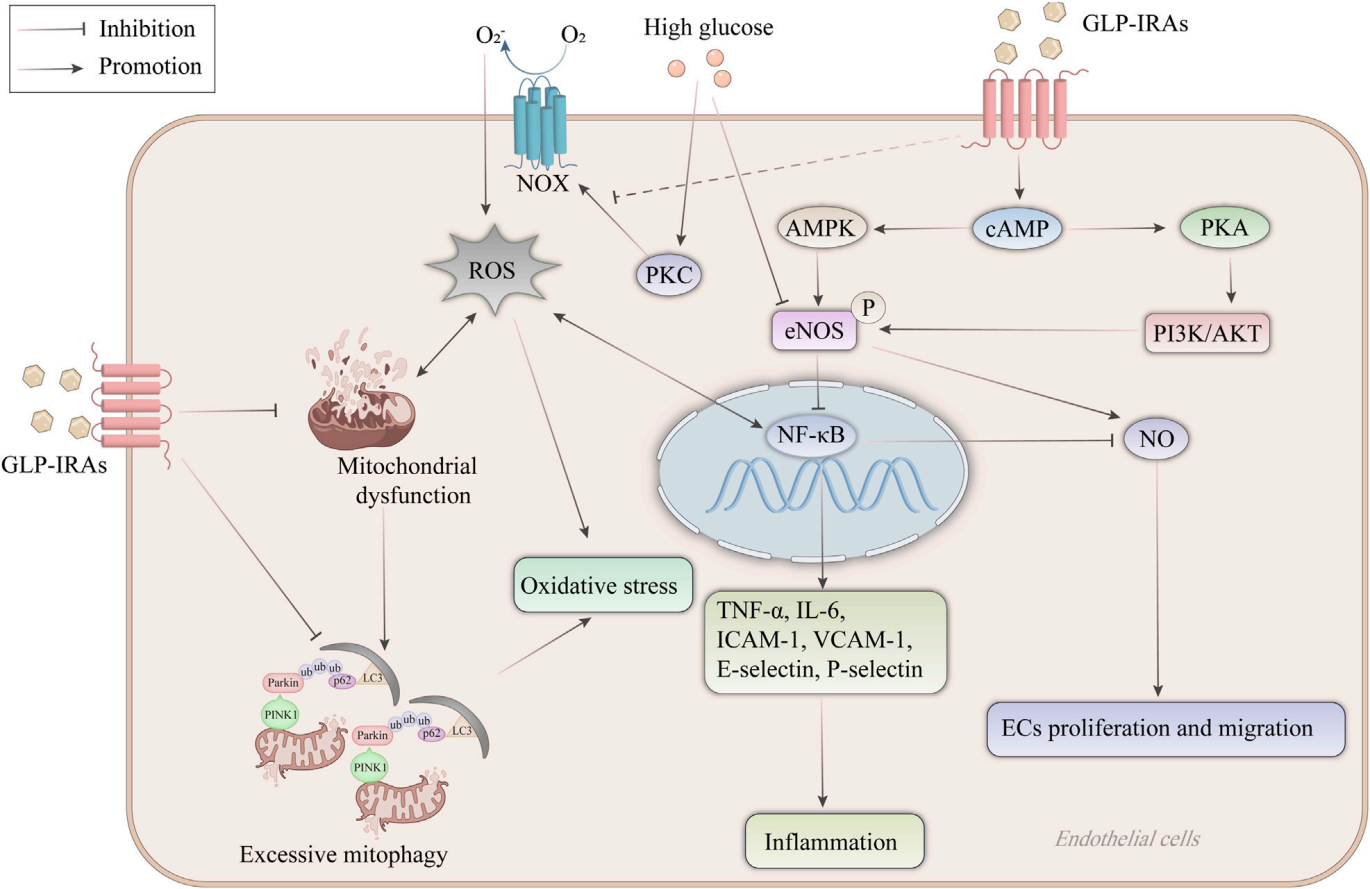
Mechanism of GLP-1RAs in protecting endothelial cell function. High glucose leads to eNOS uncoupling, and high glucose stimulates the production of ROS from NOX expressed on endothelial cells through activation of PKC. ROS lead to mitochondrial dysfunction, increase the production of NF-κB, and mediate oxidative stress in endothelial cells. NF-κB increases the expression of inflammatory factors and adhesion molecules such as TNF-α, IL-6, ICAM-1, VCAM-1, E-selectin and P-selectin, and inhibits NO synthesis in endothelial cells, thereby inducing endothelial cell proliferation and migration. GLP-1RAs activate eNOS to inhibit the NF-κB signaling pathway and increase NO synthesis through the cAMP-dependent AMPK and PI3K/AKT signaling pathways. In addition, mitophagy plays a vital role in maintaining endothelial cell homeostasis, whereas excessive mitophagy after mitochondrial dysfunction increases ROS release instead. GLP-1RAs not only ameliorated mitochondrial dysfunction but also inhibited the recruitment of PINK1/Parkin, preventing excessive mitophagy. AKT, protein kinase B; AMPK, AMP-activated protein kinase; cAMP, cyclic adenosine monophosphate; ECs, endothelial cells; eNOS, endothelial-type nitric oxide synthase; GLP-1RAs, glucagon-like peptide-1 receptor agonists; ICAM-1, intercellular adhesion molecule-1; IL-6, interleukin-6; LC3, microtubule-associated protein 1A/1B-light chain 3; NF-κB, nuclear factor-κB; NO, nitric oxide; NOX, NAPDH oxidase; PI3K, phosphatidylinositol 3-kinase; PINK1, PTEN-induced putative kinase 1; PKA, protein kinase A; PKC, protein kinase C; ROS, reactive oxygen species; TNF-α, tumor necrosis factor-α; ub, ubiquitin; VCAM-1, vascular cell adhesion molecule-1.

Endothelial cells play a crucial role in the pathophysiological process of AS. GLP-1RAs exert protective effects on endothelial cell function by inhibiting endothelial cell expression of inflammatory factors and adhesion molecules, inducing NO synthesis in endothelial cells, protecting mitochondrial function, and inhibiting oxidative stress (Figure 2).

An animal study showed that liraglutide attenuated intimal hyperplasia after coronary stent implantation and significantly lower levels of interleukin-6 (IL-6) and tumor necrosis factor-α (TNF-α), two pro-inflammatory factors and elevated IL-10, an anti-inflammatory factor, levels (Bruen et al., 2019; Berndt et al., 2021). In a meta-analysis incorporating data from SUSTAIN3 and PIONEER1, 2, and 5, semaglutide significantly reduced hypersensitive C-reactive protein levels (Rakipovski et al., 2018; Jensen J. K. et al., 2022). It is believed that inflammatory alterations in endothelial cells, caused by blood flow, occur concurrently with the development of AS. Endothelial cell activation leads to the expression of intercellular adhesion molecule-1 (ICAM-1), vascular adhesion molecules such as vascular cell adhesion molecule-1 (VCAM-1), E-selectin, and P-selectin, which draw in lymphocytes and monocytes, attach to endothelial cells, and penetrate the artery, initiating an inflammatory response (Libby, 2021). In an *in vitro* model, GLP-1RAs have been reported to downregulate these adhesion molecules’ level in endothelial cells, which coincided with a decrease in pro-inflammatory indicators such as TNF-α, IL-6, nuclear factor-κB (NF-κB), and monocyte chemotactic protein-1 (MCP-1) (Zhang et al., 2020). In addition, GLP-1RAs block oxidized LDL (ox-LDL)-induced monocyte adhesion to endothelial cells via regulating the ERK5/KLF2 signaling pathway (Yue et al., 2019). By downregulating certain inflammatory factors and adhesion molecules in the artery wall, GLP-1RAs prevent the concentration of monocytes and macrophages, leading to inflammation inhibition in the endothelial cells and achieving an anti-AS effect. Given the importance of inflammation in the development of AS, the therapeutic concept of reducing inflammation as a strategy to prevent AS and its complications has gained increasing attention, with research on GLP-1RAs contributing significantly to advancing this concept (Luna-Marco et al., 2023).

### 5.2 Induction of NO synthesis in endothelial cells

Endothelial-type nitric oxide synthase (eNOS) has been reported to have cardioprotective functions (Helmstädter et al., 2020). In in vitro experiments, GLP-1RAs have been shown to bind to GLP-1R on endothelial cells, leading to activated eNOS, increased NO production, and inhibition of NF-κB activation, thereby protecting endothelial cells (Tang et al., 2018; Wu et al., 2021). Erdogdu et al. (Erdogdu et al., 2010) found that eNOS in exenatide-stimulated human coronary artery endothelial cells (hCAECs) is activated by phosphorylation through cyclic adenosine monophosphate (cAMP)-dependent AMPK and PI3K/AKT signaling pathways, resulting in a direct protective effect on endothelial cells. Exenatide also promotes hCAEC proliferation in a MAPK/ERK1/2-independent manner (Han et al., 2019). Similarly, Wei et al. (Wei et al., 2016) confirmed Erdogdu’s report that exenatide stimulation of human umbilical vein endothelial cells (HUVECs) led to the phosphorylation of eNOS, inducing NO production, an effect that could be eliminated by GLP-1RAs inhibitors and by specific inhibitors of signaling pathways. Liraglutide enhances eNOS expression in aortic endothelial cells and increases NO production, exerting an anti-inflammatory effect in vascular endothelial cells (Durak and Turan, 2023). Also, liraglutide functions as a catalyst to promote endothelial cell migration and proliferation, directly affecting vascular endothelial repair and revascularization (Yue et al., 2019). In addition, eNOS is not only expressed in endothelial cells but also in cardiomyocytes (Xiao et al., 2020). Regulation of cardiac NO synthesis has been suggested as a common pathway to explain the benefits of multiple effective therapies for ASCVD (Aung et al., 2019).

### 5.3 Protection of mitochondrial function and inhibition of oxidative stress

Reactive Oxygen Species (ROS) are a class of oxidizing molecules with unstable electrons, such as superoxide anion, hydrogen peroxide, and hydroxyl radicals (Yan et al., 2023). While ROS facilitates the regulation of cellular signaling and gene expression at a certain level, excessive ROS causes oxidative stress, resulting in vascular endothelial dysfunction, LDL oxidation, and, ultimately, ASCVD (Yu et al., 2023). ROS are produced by various sources, including NAPDH oxidase 4 (NOX), mitochondrial electron transport chain, and eNOS uncoupling (Senoner and Dichtl, 2019). The enzyme complex NOX located in the cell membrane produces superoxide radicals from oxygen (Chen et al., 2023). Protein kinase C (PKC) can activate NOX and increase ROS production brought on by elevated glucose levels (Aimo et al., 2020). By preventing PKC-mediated activation of NOX, liraglutide decreased the generation of ROS in human aortic endothelial cells (Sivalingam et al., 2021). Mitochondria serve as the cellular powerhouses, generating adenosine triphosphate (ATP) through oxidative phosphorylation and participating in calcium regulation and apoptosis (Wang and Kang, 2020). Impairment of the mitochondrial respiratory chain or high proton motive force increases ROS production. In contrast, GLP-1RAs can attenuate oxidative stress, improve respiratory chain activity, increase ATP production, and delay the progression of AS by protecting mitochondrial function (Luna-Marco et al., 2023). The generation of ROS and the preservation of cellular homeostasis depend heavily on mitophagy, a process driven by the phosphatase and tensin homolog (PTEN)-induced putative kinase 1 (PINK1) (Guan et al., 2023). Under conditions of cellular stress, the mitochondrial membrane potential decreases, and PINK1 is unable to enter the inner mitochondrial membrane. Instead, it accumulates in the outer membrane, recruiting Parkin to activate mitophagy (Bader and Winklhofer, 2020). A cytoplasmic ubiquitin ligase called parkin migrates to injured mitochondria and promotes ROS leakage, which leads to mitochondrial breakdown (Connelly et al., 2023). However, under pathological conditions, excessive mitophagy may be a double-edged sword, especially in endothelial cells undergoing AS, where it results in mitochondrial breakage and excessive ROS production. In HUVECs, liraglutide exerts its effects at an earlier stage of this pathway by impeding the recruitment of PINK1/Parkin, reducing the occurrence of excessive mitophagy, and decreasing ROS release (Vandemark et al., 2023). A mouse cardiomyocyte model of hypoxia *in vitro* showed that exenatide treatment resulted in a decrease in mitochondrial generation of ROS in cells. In contrast, intracellular signaling related to the maintenance of mitochondrial integrity was upregulated in these cells (Lambadiari et al., 2018). In addition to fundamental studies, a short clinical experiment was conducted to evaluate the antioxidant benefits of GLP-1RAs. It showed that plasma eight-iso-prostaglandin F2α (8-iso-PGF2α) levels were reduced in individuals with T2DM after 26 weeks of weekly injections of dulaglutide, suggesting lower levels of oxidative stress (Chang et al., 2019). The studies indicate that GLP-1RAs have antioxidant properties by decreasing ROS generation, and the effects are partially independent of their glucose-lowering effects.

### 5.4 Inhibition of NLRP3 inflammasome

**FIGURE 3:**
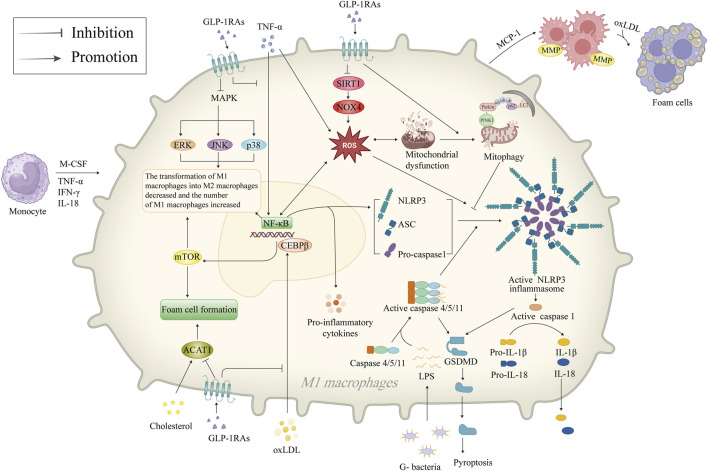
Mechanism of GLP-1RAs in reducing macrophage inflammation and foam cell formation. Inflammatory factors such as M-CSF, TNF-α, IFN-γ, and IL-18 attract monocytes to migrate and infiltrate in the vasculature of AS, differentiate into M1 macrophages, and activate the NF-κB signaling pathway, which mediates the generation of ROS. M1 macrophages accumulate and phagocytose oxLDL to become foam cells. NF-κB promotes the release of more inflammatory factors from the macrophages and activates NLRP3 inflammasome. The NLRP3 inflammasome is a crucial component of the innate immune system, inducing the secretion of pro-inflammatory cytokines IL-1β/18, activating caspase-1 to promote GSDMD-mediated cellular pyroptosis. GLP-1RAs promote mitophagy in dysfunctional mitochondria and inhibit ROS production via the SIRT1/NOX4 signaling pathway, thereby inhibiting the activation of NLRP3 inflammasome. GLP-1RAs promote the transformation of M1 macrophages to M2 through inhibition of the CEBPβ/mTOR and MAPK signaling pathways and increase the proportion of M2 macrophages with anti-inflammatory functions. In addition, GLP-1RAs directly inhibits foam cell formation by downregulating ACAT1. ACAT1, acetyl-CoA acetyltransferase 1; AS, atherosclerosis; ASC, apoptosis-associated speck-like protein with a caspase recruitment domain; CEBPβ, CCAAT/enhancer-binding proteinβ; ERK, extracellular signal-regulated kinase; G-, Gram-negative; GLP-1RAs, glucagon-like peptide-1 receptor agonists; GSDMD, gasdermin D; IFN-γ, interferon-γ; IL-18, interleukin-18; IL-1β, interleukin-1β; JNK, c-Jun N-terminal kinase; LC3, microtubule-associated protein 1A/1B-light chain 3; MAPK, mitogen-activated protein kinase; MCP-1, monocyte chemotactic protein-1; M-CSF, macrophage colony-stimulating factor; MMP, matrix metalloproteinase; mTOR, mammalian target of rapamycin; NF-κB, nuclear factor-κB; NLRP3, NOD-like receptor thermal protein domain associated protein 3; NOX4, NAPDH oxidase four; oxLDL, oxidized low-density lipoprotein; PINK1, PTEN-induced putative kinase 1; ROS, reactive oxygen species; SIRT1, sirtuin 1; TNF-α, tumor necrosis factor-α; ub, ubiquitin.

Macrophages are present throughout the development of AS, contributing to the formation of foam cells after phagocytosis of ox-LDL and further releasing inflammatory cytokines. GLP-1RAs have been reported to inhibit macrophage NLRP3 inflammasome, reducing macrophage inflammation and foam cell formation, highlighting GLP-1RAs’ vital protective role in AS (Figure 3).

NLRP3 is a 118 kDa cytoplasmic pattern recognition receptor (PRR) protein found in a wide type of cells (Ye et al., 2023; Zhang et al., 2023). The cytoplasmic multiprotein signaling complexes, known as NLRP3 inflammasomes, are formed when NLRP3 is activated (Wang and Hauenstein, 2020). The caspase-1, apoptosis-associated speck-like protein with a caspase recruitment domain (ASC), and the NLRP3 protein make up the NLRP3 inflammasome (Li et al., 2020a). Hyperactivation of the NLRP3 inflammasome is linked to the pathogenic progression of ASCVD (Abbate et al., 2020). Duewell et al. (Tanase et al., 2023) demonstrated the NLRP3 inflammasome’s critical role in AS by using LDL receptor (LDLR)-deficient mice with NLRP3^−/−^, ASC^−/−^, and IL-1α/β−/− bone marrow. After 8 weeks of high-fat diets, mice showed reduced levels of IL-18, caspase-1/11 in the bone marrow, and significantly reduced AS plaques (Liaqat et al., 2020), providing the first solid evidence that NLRP3 inflammasome promotes AS development. Following this observation, Shi et al. (Xu et al., 2022) found upregulated levels of NLRP3, ASC, caspase-1, IL-1β, and IL-18 in carotid atherosclerotic plaques from patients undergoing carotid endarterectomy, with unstable plaques expressing higher levels of these compounds as opposed to stable plaques. In contrast, inhibition of the NLRP3 signaling pathway by siRNA interference suppressed pro-inflammatory cytokines in high-fat diet-fed ApoE^−/−^ mice (Jin et al., 2022). Given the critical role of the NLRP3 inflammasome in the onset of AS, using NLRP3 inflammasome as therapeutic targets has become a new hotspot in anti-AS drug research (Martínez et al., 2018). Additionally, GLP-1RAs may be related to the NLRP3 inflammasome. Liraglutide, for example, was shown to reverse sirtuin 1 (SIRT1), NOX4 levels, and ROS generation by modulating TNF-α and SIRT1/NOX4/ROS pathway in the hypoxia-induced H9c2 cells, thereby suppressing the NLRP3 inflammasome-dependent cellular pyroptosis. In contrast, the SIRT1 inhibitor EX 527 nullified the beneficial impact of liraglutide (Chen et al., 2018; Luo et al., 2019). Song et al. (Song et al., 2022) also demonstrated the impact of liraglutide in attenuating central nervous inflammation and demyelination was also associated with the modulation of the NLRP3 pathway. In addition, GLP-1RAs inhibit NLRP3 inflammasome both directly and indirectly. Shao et al. (Wang W. et al., 2023) found that GLP-1RAs mitigate liver damage caused by oxidative stress by promoting the mitophagy pathway and suppressing the NLRP3 inflammasome, an effect that can be eliminated by PINK1 siRNA.

### 5.5 Reduction of macrophage inflammation and foam cell formation

Under pathological conditions, macrophages participate in the process of inflammation, which drives the progression of AS (Linton et al., 2019). GLP-1RAs reduce macrophage infiltration and inhibit macrophage-derived inflammatory factors (Yang et al., 2021). Animal studies have shown that semaglutide slowed plaque progression in rabbits with AS compared to the placebo group, and the histologic examination showed decreased macrophage infiltration despite there being lower LDL levels in the control group (Pan et al., 2023). Similar results were observed in ApoE^−/−^ mice treated with liraglutide (Jensen D. M. et al., 2022). An *in vitro* study has demonstrated that treatment with liraglutide reduced MCP-1 levels, and macrophage TNF-α and IL-1β secretion in AS plaques and peripheral blood macrophages from patients with human carotid endarterectomy (Zheng et al., 2019). In addition, GLP-1RAs promote macrophage polarization toward M2, decrease the number of pro-inflammatory macrophages, and attenuate AS plaque load in the aortic root of ApoE^−/−^ mice by affecting the CCAAT/enhancer-binding protein β, the MAPK and NF-κB signaling pathways (Li et al., 2020b). A clinical study conducted by Yang et al. (Yang et al., 2021) further demonstrated a reduction in the overall count of macrophages, an elevation in the percentage of M2 macrophages in the peripheral blood, and higher cell surface GLP-1R expression among individuals suffering from coronary artery disease by contrast with the controls. The increased percentage of M2 macrophages with anti-inflammatory and enhanced function decreased harmful alterations to the artery wall and promoted the stabilization of AS lesions (Hou et al., 2023). Thus, given the methods mentioned above, GLP-1RAs may prevent inflammation linked with macrophages. Foam cells, primarily derived from macrophages and VSMCs, constitute an essential component of AS plaques (Shah, 2019). Tashiro et al. (Tashiro et al., 2014) demonstrated that liraglutide inhibits the phenotypic conversion of VSMCs to macrophages, decreasing the generation of foam cells as well as the plaque area, and ultimately retarding the growth rate of AS plaques. A subsequent animal study revealed that liraglutide could inhibit foam cell formation by downregulating acetyl-CoA acetyltransferase 1 (ACAT1) (Qin and Song, 2022). This aligns with the findings of earlier research and fully demonstrates the direct inhibitory effect of GLP-1RAs on AS plaque formation.

### 5.6 Improvement of VSMC dysfunction

**FIGURE 4:**
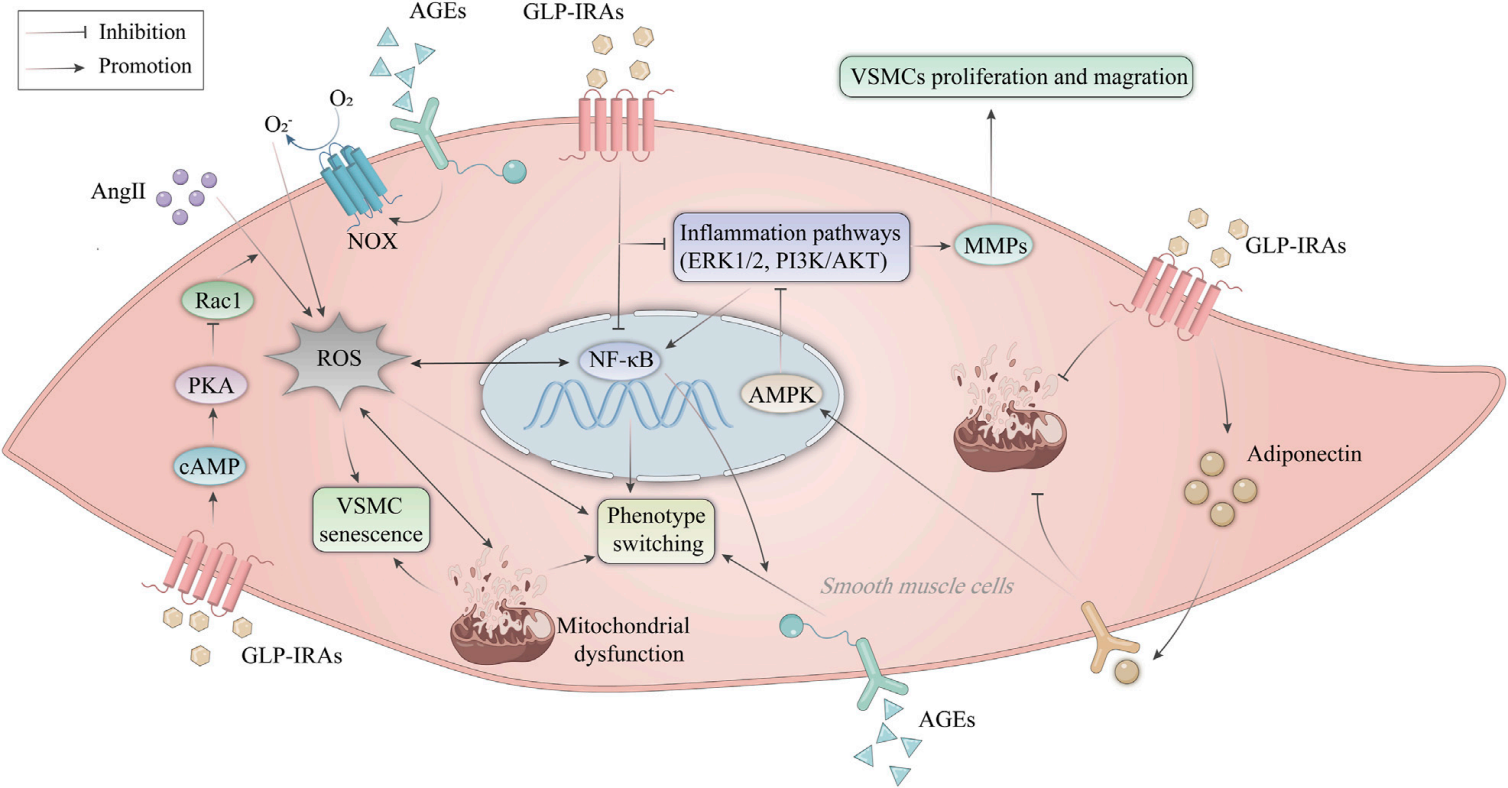
Mechanism of GLP-1RAs in improving VSMC dysfunction. AngII promotes ROS production in VSMCs and activates the NF-κB signaling pathway. AGEs activate NOX expressed on endothelial cells to produce ROS. ROS leads to mitochondrial dysfunction, inducing phenotype switching and premature senescence of VSMCs. GLP-1RAs inhibit the activation of Rac1 via the cAMP/PKA pathway, thereby resisting Ang II-induced ROS production and corresponding VSMC senescence. In addition, GLP-1RAs inhibit VSMC proliferation and migration by suppressing inflammatory signaling pathways such as NF-κB, ERK1/2, and PI3K/AKT. Activation of AMPK by APN inhibits the ERK1/2 and PI3K/AKT signaling pathways and attenuated mitochondrial dysfunction. GLP-1RAs increases the expression of APN in VSMCs. AGEs, advanced glycation end products; AKT, protein kinase B; AMPK, AMP-activated protein kinase; AngII, angiotensin II; APN, adiponectin; cAMP, cyclic adenosine monophosphate; ERK, extracellular signal-regulated kinase; GLP-1RAs, glucagon-like peptide-1 receptor agonists; MMPs, matrix metalloproteinases; NF-κB, nuclear factor-κB; NOX, NAPDH oxidase; PI3K, phosphatidylinositol 3-kinase; PKA, protein kinase A; Rac1, Ras-related C3 botulinum toxin substrate 1; ROS, reactive oxygen species; VSMCs, vascular smooth muscle cells.

VSMC dysfunction is defined as abnormal proliferation, migration, senescence, apoptosis, necrosis, and phenotypic transformation (Zhu et al., 2019; Wang Q. et al., 2023), leading to aggravated vascular damage and accelerated progression of AS. Aberrant VSMC proliferation and migration promote the development of necrotic cores and the thickness of AS plaques (Miano et al., 2021). Here, we will discuss the role of GLP-1RAs in improving VSMC dysfunction (Figure 4).

Liraglutide was reported by Shi et al. (Zhou et al., 2023) to inhibit high-glucose-induced aberrant proliferation and migration of VSMCs through the ERK1/2 and PI3K/AKT signaling pathways (Allahverdian et al., 2018). While impaired VSMC autophagy accelerates stress-induced early senescence of VSMCs, activated autophagy helps VSMCs withstand toxic damage and support cell survival (Grootaert et al., 2018). Therefore, well-functioning VSMCs are essential to protect the vessel wall from AS. Numerous factors, including DNA damage, telomere shortening, oxidative stress, and mitochondrial dysfunction, may cause VSMC senescence (Chi et al., 2019; Shi et al., 2020). As a result of senescent cells’ diminished capacity for proliferation, the fibrous cap VSMC content decreases, which disrupts repair after plaque rupture and promotes plaque instability (Durham et al., 2018). Senescence is a stress-adapted state in which VSMCs stop proliferating, undergoing significant morphological and metabolic changes while remaining alive. The cells choose to die when the harmful effects become too intense or too long-lasting (Bonetti et al., 2021). Exenatide has been shown to inhibit the activation of Ras-related C3 botulinum toxin substrate 1(Rac1) via the cAMP/PKA pathway, thereby resisting the generation of superoxide and subsequent VSMC aging induced by Angiotensin II (Ang II) (Berndt et al., 2021). The quiescent contractile and differentiable phenotypes of VSMC give way to the activated synthetic and dedifferentiated phenotypes during the phenotypic shift (Ashraf and Al Haj Zen, 2021). In rat coronary VSMCs, liraglutide blocks advanced glycation end products (AGEs)-induced phenotypic transformation via suppressing the NF-κB signaling pathway and activating the cAMP/PKA signaling pathway (Di et al., 2019). In addition, exenatide regulates mitochondrial dynamics by promoting VSMC adiponectin (APN) expression and attenuates mitochondrial dysfunction, thereby inhibiting VSMC dedifferentiation (Fan et al., 2020). Therefore, GLP-1RAs can reverse vascular remodeling and delay VSMC senescence, and investigating the molecular pathways behind VSMC dysfunction might provide evidence for developing novel approaches and finding therapeutic targets for managing ASCVD (Khat and Husain, 2018).

## 6 Conclusion

Clinical trials and mechanistic studies have demonstrated that GLP-1RAs offer favorable cardiovascular benefits independent of their known effects on glucose metabolism. These benefits may directly improve endothelial, VSMC, and immune cell function (Iorga et al., 2020). However, further investigation is required to ascertain whether this mechanism of cardiovascular protection is influenced by the activation of GLP-1R in the local area or by the cumulative peripheral effects that indirectly enhance vascular function. Additionally, the selection of GLP-1RAs for particular patients should take into account the structural variations amongst various GLP-1RAs since these variations may result in distinct clinical presentations. Subsequent clinical studies should explore how GLP-1RAs work in concert with the current standard care for ASCVD. In summary, continuously improving the molecular mechanism of GLP-1RAs in treating ASCVD will provide valuable insights for treating ASCVD.
